# Encapsulation and Characterization of Proanthocyanidin Microcapsules by Sodium Alginate and Carboxymethyl Cellulose

**DOI:** 10.3390/foods13050740

**Published:** 2024-02-28

**Authors:** Yanfei Li, Huan Zhang, Yan Zhao, Haoxin Lv, Kunlun Liu

**Affiliations:** 1 Food Engineering Technology Research Center/Key Laboratory of Henan Province, Henan University of Technology, Zhengzhou 450001, China; liyanfei@haut.edu.cn (Y.L.); lvhaoxin0129@126.com (H.L.); knlnliu@126.com (K.L.); 2 School of Food and Strategic Reserves, Henan University of Technology, Zhengzhou 450001, China; zhanghuan9257@163.com

**Keywords:** proanthocyanidins, microcapsules, characterization, stability

## Abstract

Proanthocyanidins are important compounds known for their antioxidant and radical scavenging properties, but they are highly sensitive to light, heat, oxygen, and pH. In our study, proanthocyanidin was encapsulated using sodium alginate and carboxymethyl cellulose to enhance controlled release, pH stability, metal ion tolerance, temperature resistance, time release, the microencapsulation of food additives stability, antioxidant capacity analysis, and the storage period tolerance of proanthocyanidin. Fourier transforms infrared (FTIR) analysis and full-wavelength UV scanning indicated the successful immobilization of proanthocyanidins into the polymeric microcapsules. The flowability and mechanical properties of the microcapsules were enhanced. Moreover, proanthocyanidin microcapsules exhibited higher thermal, pH, metal ion, time, and microencapsulation food additive stability. In addition, due to their high antioxidant properties, the proanthocyanidin microcapsules retained a greater amount of proanthocyanidin content during the gastric phase, and the proanthocyanidin was subsequently released in the intestinal phase for absorption. Thus, the study provided a systematic understanding of the antioxidant capabilities and stability of proanthocyanidin microcapsules, which is beneficial for developing preservation methods for food additives.

## 1. Introduction

Proanthocyanidins (PC), also known as condensed tannins, are a group of natural bioflavonoid compounds formed via the condensation of flavan-3-ol structural units [1,2,3]. Natural PC is commonly found in a variety of plants, such as grapes, cocoa, apples, blueberries, hawthorn, raspberries, and beans, primarily in their skins, cores, and stalks [4], with higher concentrations in plant tissues [5]. Proanthocyanidin molecules contain multiple phenolic hydroxyl structures that can provide hydrogen atoms to neutralize free radicals and competitively bind to them, effectively interrupting free radical chain reactions. Additionally, the semiquinone radicals produced by these reactions can undergo nucleophilic addition reactions to form catechins and pyrogallol structures with potent antioxidant activity [6]. This process inhibits inflammation and the development of cardiovascular and cerebrovascular diseases caused by free radicals. However, PC exhibits lower stability and is vulnerable to oxygen, light, enzymes, temperature, metal ions, and oxidants during food processing [7]. Pereira et al. [8] suggested that the presence of acidic phenolic hydroxyl groups and unsaturated bonds in the molecular structure of PC results in their limited long-term storage stability and makes them susceptible to degradation, oxidation, and polymerization. Phenolic compounds in the presence of strong oxidants can lead to the oxidative degradation of PC [9].

The use of microencapsulation technology helps protect unstable substances, such as polyphenols, which are prone to degradation. Sodium alginate is a natural, non-toxic macromolecular substance composed of α-L-guluronic acid (G) and β-D-mannuronic acid (M). It is considered an ideal encapsulation material, but single sodium alginate has certain limitations [10,11]. This has prompted the incorporation of carbohydrate polymers to enhance the chemoprotective properties and stability of sodium alginate microcapsules in gastric conditions [12]. Carboxymethyl cellulose, a water-soluble, chemically modified natural cellulose with numerous reactive groups such as carboxyl and hydroxyl, can form complexes and coordinate with metal ions to enhance the mechanical strength of hydrogels [13]. This compound demonstrates excellent physical and chemical properties and can partially offset the mechanical property deficiencies of sodium alginate microcapsules. Sheng et al. [14] used a blend of sodium alginate, methylcellulose, and hydroxypropyl methylcellulose to encapsulate grape seed PC. The researchers found that the composite wall material showed greater thermal stability compared to the individual wall materials. Nwabor et al. [15] combined sodium alginate and carboxymethyl cellulose to synthesize eucalyptus polyphenol microcapsules. They achieved encapsulation rates ranging from 74% to 82%, demonstrating favorable encapsulation efficacy. The preparation of sodium alginate microcapsules commonly involves methods such as spray drying, extrusion, emulsion gelation, and layer assembly [16]. Among these, the sharp pore method is considered the simplest approach for producing sodium alginate microcapsules with uniform particle size [17]. The resulting microcapsules demonstrate relatively high stability in simulated gastric and simulated intestinal fluids, attributed to the coordination of carboxyl and hydroxyl groups of every four G monomers with metal cations, particularly sodium and calcium ions, leading to the formation of a tight “eggshell” model [18,19].

In this study, proanthocyanidin microcapsules were prepared by the sharp-pore method using sodium alginate and carboxymethyl cellulose as wall materials, to provide a theoretical basis and technical support for the various utilization of PC.

## 2. Materials and Methods

### 2.1. Materials and Reagents

PC (molecular weight: 9594.52), carboxymethylcellulose (CMC) (molecular weight: 90.0778), and calcium chloride (molecular weight: 110.98) were purchased from Maclean Biochemical Technology Co., Ltd. (Shanghai, China). Sodium alginate (molecular weight: 216.121), diammonium salt (ABTS), (molecular weight: 548.68), DPPH (molecular weight: 394.32), Pepsin (CAS: 9001-75-6), potassium sorbate (molecular weight: 150.218), sodium benzoate (molecular weight: 144.105), citric acid (molecular weight: 192.12), and sodium bisulfite (molecular weight: 104.061) were obtained from Qianzhi Trading Co., Ltd. (Henan, Zhengzhou, China).

### 2.2. Preparation of Microcapsules

Proanthocyanidin microcapsules were prepared using the method described by Nwabor et al. [15] with slight modifications. Proanthocyanidin microcapsules were prepared using procyanidin powder as the core material. A solution of a 1:1 compound of sodium alginate and carboxymethyl cellulose solution and calcium chloride was used as the wall material, and a solution of calcium chloride was used as the curing solution. The core material was added to the wall solution in a specific proportion, stirred to mix, and sonicated at 120 W and 25 °C to enhance dissolution and eliminate air bubbles. The mixture was aspirated into a syringe and then dispensed into a calcium chloride solution at a rate of 0.25 drops per second from a height of 8 cm, cured with slow stirring for 3 h, and subsequently filtered through extraction to obtain wet microcapsules. The wet microcapsules obtained were freeze-dried for 48 h to obtain lyophilized microcapsules.

### 2.3. Calculation of Encapsulation Rate

The microcapsules containing the calcium chloride solution were extracted and filtered. Subsequently, the microcapsules were rinsed with distilled water, the filtrate was combined, and the proanthocyanidin content was indirectly measured using the n-butanol-hydrochloric acid method.

(1) $$R=\frac{m1-m2}{m1}\times 100\%$$

Note: R is the microencapsulation rate of PC, %; m1 is the total content of PC at the beginning, mg; and m2 is the residual content of PC in the filtrate, mg.

### 2.4. Basic Physical Properties

#### 2.4.1. Apparent Morphology

Proanthocyanidin microcapsules in wet and freeze-dried states were photographed using a camera. Microcapsule images were obtained by observing the freeze-dried microcapsule morphology under a light microscope at 40× magnification [19].

#### 2.4.2. Grain Size

The particle size was measured using a digital vernier caliper, 50 readings were recorded from randomly selected wet and lyophilized microcapsules, and finally, their average values were calculated as the experimental results.

#### 2.4.3. Bulk Density and Compact Density

For the determination of bulk density [20], a certain mass of lyophilized microcapsules was loaded into a measuring cylinder, and the cylinder was shaken horizontally and uniformly to make the product settle naturally; the volume of the sample was measured at this time, and the bulk density was calculated by Equation (2).

(2) $$\mathrm{Bulk}\mathrm{density}\;(\rho 1)=\frac{m}{v}$$

The compact density was assessed by gently tapping the measuring cylinder 50 times to bring the microcapsules to a compact state based on the stacked density, recording the cylinder readings, and calculating the compact density from Equation (3) [21].

(3) $$\mathrm{compact}\mathrm{density}\;(\rho 2)=\frac{m}{v}$$

Note: m is the mass of the weighed microcapsules, g; and v is the corresponding volume, mL.

#### 2.4.4. Flowability

The mobility of microcapsules was assessed using a combination of the angle of repose, the Carr index (CI), and the Hausner ratio (HR). The angle of repose was measured using the injection method: the lyophilized sample was slowly added from the top of the funnel and was allowed to fall naturally into the funnel, forming a pile up on the circular plate. The angle of repose of the sample was calculated using Equation (4). The CI and HR were calculated by Equations (5) and (6).

(4) $$\mathrm{Angle}\mathrm{of}\mathrm{repose}\;(\beta )=\frac{\mathrm{arctanH}}{r}$$

Note: H is the height of the lyophilized microcapsules, mm; and r is the corresponding microcapsule stack radius, mm.

(5) $$\mathrm{CI}\;=\frac{\rho 2-\rho 1}{\rho 2}\times 100$$

(6) $$\mathrm{HR}=\frac{\rho 2}{\rho 1}$$

Note: ρ1 is the bulk density, g/mL; and ρ2 is the compact density, g/mL.

### 2.5. Color Difference

The colors of the microcapsules, PC, sodium alginate, and carboxymethyl cellulose were measured using a color difference meter and recorded as L*, a*, and b*. L* indicates the brightness in the range of 0~100 from black to white, a* indicates the brightness from red (+) to green (−), and b* indicates the brightness from yellow (+) to blue (−). The instrument was standardized with a standard white plate before measurement, and the measurement was repeated three times. Chroma, Hue angle (Hue), and Browning (BI) were calculated from Equations (7)–(9) [21].

(7) $$\mathrm{Chroma}=\sqrt{{a*}^{2}+{b*}^{2}}$$

(8) $${\mathrm{Hue}=\tan}^{-1}\left(\frac{b*}{a*}\right)$$

(9) $$\mathrm{BI}=\frac{100(0.31X)}{0.17},\;X=\frac{a*+1.75L*}{5.645L*+a*-3.012b*}$$

### 2.6. Texture

A mass spectrometer was used to analyze the full mass spectra of the unfreeze-dried microcapsules. Conditions: the TPA item was selected, probe speed was set at 1.0 mm/s before, during, and after measurement, the starting force was set to Auto 5.0 g, the compression distance was 50%, and the time was 5 s. Data were collected to compare the hardness, elasticity, cohesiveness, gumminess, and chewiness of microcapsules. Each sample was measured five times, and the values were expressed as the mean ± standard deviation.

### 2.7. Thermogravimetric Analysis

Small amounts of PC, microcapsules, and wall materials were taken into small aluminum crucibles for thermogravimetric analysis [22]. The measured temperature range was from 30 to 500 °C, and the heating rate was 10 °C/min. Liquid nitrogen was used as the cooling substance, and the heating process was carried out with Kor pure nitrogen as the purge gas and protective gas.

### 2.8. UV-Vis Analysis

A certain amount of PC, microcapsules, and wall materials were weighed and dissolved in distilled water, the UV full wavelength scan of 200~800 nm was carried out with the distilled water as the baseline, and the scan interval was 0.5 nm.

### 2.9. Fourier Infrared Spectroscopy

The spectra of PC, microcapsules, and wall materials were measured by FTIR analysis. Samples were ground with potassium bromide under a certain pressure to compress into thin film layers. Each spectrum is the result of 64 scans at 4 cm^−1^ resolution. The spectra were obtained in the region of 400 to 4000 cm^−1^. All readings were taken at room temperature (20 °C).

### 2.10. Stability

#### 2.10.1. pH Stability Determination

A total of 20 mg of microcapsules (or PC) was mixed with 20 mL of phosphate-buffered solution of different pH (2, 4, 6, 8, and 10) for 30 min. The mixture was filtered and centrifuged at 5000 rpm for 10 min. An amount of 70 μL of supernatant and 210 μL of DMAC solution were mixed, and the absorbance rate at 420 nm wavelength was determined. The initial amount of PC was recorded as A0, and the post-storage content of proanthocyanins in microcapsules was recorded as A. The retention rate of anthocyanins was calculated according to Equation (10).

(10) $$\mathrm{Proanthocyanidin}\;\mathrm{retention}\;\mathrm{rate}=(\frac{A0-A}{A0})\times 100\%$$

Note: A0 is the initial amount of PC, mg; and A is the measured proanthocyanidin content, mg.

#### 2.10.2. Determination of Temperature Stability

A total of 20 mg of microcapsules (or PC) was mixed with 20 mL of distilled water and dissolved at different temperatures (10, 30, 50, 70, and 90 °C), and the supernatant was taken by centrifugation after 20 min. An amount of 70 μL of supernatant and 210 μL of DMAC solution were mixed, and the absorbance rate at 420 nm wavelength was determined. The initial amount of PC was recorded as A0, and the post-storage content of proanthocyanins in microcapsules after stored at different temperatures was recorded as A. The retention rate of anthocyanins was calculated according to Equation (10).

#### 2.10.3. Storage Stability Determination

A total of 20 mg of microcapsules (or PC) and 20 mL of distilled water were mixed under 4 °C storage at 0, 3, 7, 14, 21, and 28 days. The mixture was filtered and centrifuged at 5000 rpm for 10 min. An amount of 70 μL of supernatant and 210 μL of DMAC solution were mixed, and the absorbance rate at 420 nm wavelength was determined. The PC content was calculated from the standard curve. The initial amount of PC was recorded as A0, and the post-storage content of proanthocyanins in microcapsules was recorded as A. The retention rate of anthocyanins was calculated according to Equation (10).

#### 2.10.4. Metal Ion Stability

A total of 20 mg of microcapsules (or PC) was mixed with 20 mL of 0.01 mol/L salt solutions of different metal ions (Na^+^, Zn^2+^, Mg^2+^, K^+^, Ca^2+^, Fe^2+^, and Fe^3+^) for 30 min, respectively, and the supernatants were taken by centrifugation every 30, 60, 90, 120, 150 and 180 min. An amount of 70 μL of supernatant and 210 μL of DMAC solution were mixed, and the absorbance rate at 420 nm wavelength was determined. The PC content was calculated from the standard curve. The initial amount of PC was recorded as A0, and the post-storage content of proanthocyanins in microcapsules was recorded as A after 30, 60, 90, 120, 150, and 180 min. The retention rate of anthocyanins was calculated according to Equation (10).

#### 2.10.5. Determination of Food Additive Stability

A total of 20 mg of microcapsules (or PC) was mixed with 20 mL of potassium sorbate, sodium benzoate, citric acid, and sodium bisulfite solutions at different concentrations (0.1%, 0.2%, 0.3%, 0.4%, and 0.5%), respectively. After centrifugation, the supernatant was taken after 30 min. An amount of 70 μL of supernatant and 210 μL of DMAC solution were mixed, and the PC content was calculated by determining the absorbance rate at 420 nm wavelength. The initial amount of PC was recorded as A0, and the post-storage content of proanthocyanins in microcapsules was recorded as A after 30 min at different food additive concentrations. The retention rate of anthocyanins was calculated according to Equation (10).

### 2.11. Antioxidant Activity

#### 2.11.1. DPPH Free Radical Scavenging Ability

A DPPH solution of 0.1 mmol/L was prepared using anhydrous ethanol and stored away from light. Overall, 0.5 mL of PC equal amounts of microcapsule solution and 2 mL of DPPH solution were added to the test tubes, mixed, and then left in the dark for 30 min, and the absorbance values were measured at 515 nm. The free radical scavenging rate was calculated from Equation (11).

(11) $$\mathrm{DPPH}\;\mathrm{free}\;\mathrm{radical}\;\mathrm{scavenging}\;\mathrm{rate}=(1-\frac{A2-A1}{A0})\times 100\%$$

Note: A0 is the absorbance of distilled water instead of the sample, A1 is the absorbance of anhydrous ethanol instead of DPPH, and A2 is the absorbance of the sample.

#### 2.11.2. ABTS Free Radical Scavenging Ability Study

A 7 mmol/L ABTS solution and a 2.45 mmol/L potassium persulfate solution were prepared and mixed in equal volumes for 12 h in the dark to produce ABTS radicals, and then the ABTS solution was diluted with anhydrous ethanol to give an absorbance value of 0.70 ± 0.02 at 734 nm. Overall, 50 μL of the diluted mixture was mixed with 3 mL ABTS solution. The reaction was carried out at 25 °C for 10 min under light protection, and then the absorbance was measured at 734 nm. The free radical scavenging rate was calculated from Equation (12).

(12) $$\mathrm{ABTS}\;\mathrm{free}\;\mathrm{radical}\;\mathrm{scavenging}\;\mathrm{rate}=(1-\frac{A2-A1}{A0})\times 100\%$$

Note: A0 is the absorbance of distilled water instead of the sample, A1 is the absorbance of anhydrous ethanol instead of ABTS, and A2 is the absorbance of the sample.

### 2.12. In Vitro Digestion

Referring to the method of Munoz et al. [23] with slight modifications, the simulated gastric solution was taken as 0.2 g sodium chloride, 0.7 mL concentrated hydrochloric acid, fixed to 100 mL, and pH adjusted to 1.2. Overall, 0.2 g of microcapsules and equal amounts of PC were added to 30 mL of simulated gastric solution, 0.128 g of pepsin was added, and a constant temperature shaker was used at 37 °C for 40 r/min. And 1 mL of the cumulative release rate of PC was measured at 30, 60, 90, 120, 150, and 180 min, while 1 mL of simulated gastric juice was supplemented, and the pH of the mixture was adjusted to 7.0 at the end of the gastric digestion phase to terminate gastric digestion; the cumulative release rate of PC was calculated by Equation (13).

(13) $$\mathrm{The}\;\mathrm{cumulative}\;\mathrm{release}\;\mathrm{rate}\;\mathrm{of}\;\mathrm{PC}=\left(\frac{A}{A0}\right)\times 100\%$$

Note: A0 is the initial amount of PC, mg; and A is the experimentally measured amount of PC, mg.

The simulated intestinal digestion was performed by taking the solution digested by the stomach and adding bile salt solution (0.188 g bile salt dissolved in 4 mL of 5 mM/L phosphate-buffered solution), 1 mL of 11% calcium chloride solution, and 1 mL trypsin solution (0.145 g trypsin dissolved in 2.5 mL of 5 mM/L phosphate-buffered solution), adjusting the pH to 6.8 with sodium hydroxide solution and shaking at 37 °C for 40 rpm; 1 mL of the solution was taken to measure the proanthocyanidin content at 30, 60, 90, 120, 150 and 180 min, while 1 mL of the solution was replenished each time. Finally, the digestion mixture was heated at 95 °C for 5 min to terminate the intestinal digestion, and the cumulative release rate of PC was calculated by the same Equation (13).

### 2.13. Microencapsulation Release Kinetics

Most studies have shown that microcapsule release kinetics by Avrami’s equation [24]. The release of proanthocyanidin microcapsules at different pH (4, 6, and 8) and temperatures (4 °C, 25 °C, and 37 °C) was fitted to Avrami’s Equation (14), scatter plots were plotted, and the release rate constant k was calculated from Equation (14).

(14) R=exp[(kt)n]×100%

Note: R is the retention of PC, %; t is the time, min; n is the parameter of the release mechanism; and k is the release rate constant.

### 2.14. Data Analysis

All data are expressed as mean ± standard deviation (SD) with one-way analysis of variance and conducted in triplicate. Statistical analysis was performed using Origin 2021. Duncan’s multiple range test was conducted for significance at the *p* < 0.05 level.

## 3. Results and Analysis

### 3.1. Basic Physical Properties

#### 3.1.1. Apparent Morphology

**Figure 1:**
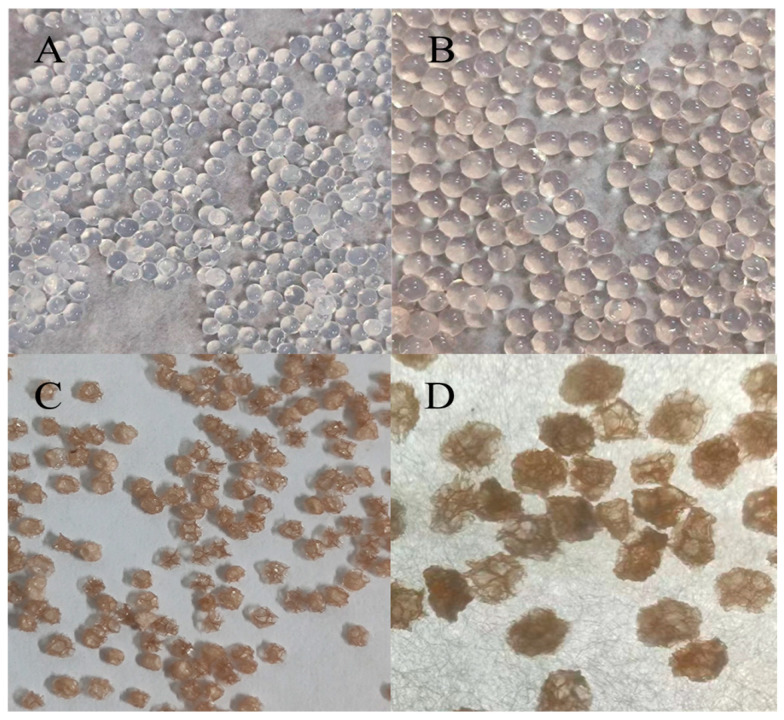
Microcapsule morphology. (**A**) The wet state blank microcapsules under optical observation. (**B**) The wet state proanthocyanidin microcapsules. (**C**) The freeze-dried proanthocyanidin microcapsules. (**D**) The freeze-dried proanthocyanidin microcapsules with 40-time magnification.

As shown in Figure 1, the blank wet-state microcapsules were transparent and colorless, while the wet-state proanthocyanidin microcapsules and the freeze-dried proanthocyanidin microcapsules showed a reddish-brown color. This coloration was a result of the loading of PC and exhibited the characteristic color of PC; Figure 1A,B exhibited that the wet state proanthocyanidin microcapsules were spherical with complete particles, and the surface was smooth without trailing and adhesion phenomenon, while in Figure 1C,D, the water evaporation in the lyophilized proanthocyanidin microcapsules exhibited a granular structure.

#### 3.1.2. Particle Size

**Figure 2:**
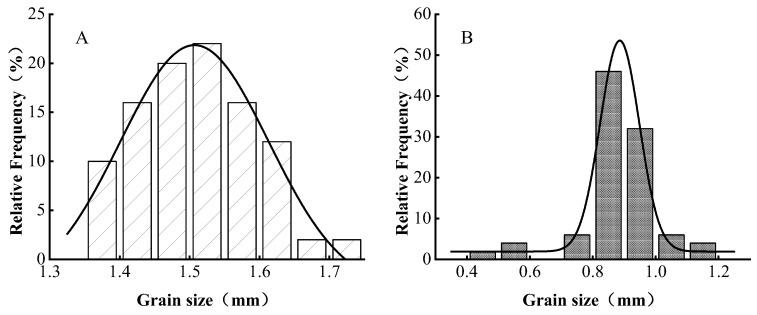
Particle size distribution of proanthocyanidin microcapsules. (**A**) The particle size distribution of proanthocyanidins in a wet state. (**B**) The particle size distribution of lyophilized microcapsules.

The size of microcapsules is generally in the range of 1 to 1000 μm; beyond this range, uneven particle size with limitations may arise [25], which is not conducive to product application. Therefore, particle size analysis was conducted on both proanthocyanidin wet microcapsules and lyophilized microcapsules. The average particle size of proanthocyanidin microcapsules in a wet state was 1.508 mm, whereas the average particle size of freeze-dried proanthocyanidin microcapsules was 820 μm, falling within the range of suitable particle sizes for microcapsules (Figure 2). Furthermore, the particle size distribution of the wet and freeze-dried proanthocyanidin microcapsules exhibited a normal distribution, with the freeze-dried proanthocyanidin microcapsules showing a narrow peak size range. This suggests that the particle size of the microcapsules was both uniform and concentrated.

#### 3.1.3. Stacking Density, Compact Density

The basic physical properties of microcapsules are shown in Table 1. Bulk density is the amount of product that can be put into a container per unit volume, and it is often used by laboratories to assess the quality parameters of microcapsules. The stacking density of lyophilized proanthocyanidin microcapsules was evaluated and the stacking density of microcapsules was obtained as 0.28 g/mL. In industrial applications, a higher bulk density could minimize the exposure of microcapsules to air, leading to reduced packaging material usage and extended shelf life. The bulk density of the microcapsules was determined to be 0.32 g/mL.

#### 3.1.4. Flowability

Flowability is often used as a quality parameter to evaluate microcapsules, and the relationship between rest angle, CI, HR, and the flowability of microcapsules is shown in Table 2. The average resting angle of the lyophilized proanthocyanidin microcapsules was 29.87° on average, and the resting angle was under 30°, which showed that the microcapsules have good mobility. The CI, measured by the bulk density and compact density, was 13.11, and the HR was 1.15, suggesting excellent mobility of the microcapsules.

### 3.2. Color Difference Analysis

The color of food materials is a significant sensory attribute that influences consumer preferences and product acceptance [26]; therefore, the color differences between proanthocyanidin microcapsules and single PC were explored. The color variation in microcapsules versus single PC and their wall materials is shown in Table 3, where L* denotes brightness, a* denotes red (+) and green (−), and b* denotes yellow (+) and blue (−). As shown in Table 3, the microcapsules exhibited significantly higher L* and b* values and lower a* values, indicating that the proanthocyanidin microcapsules originated from the wall material of the microcapsules. Furthermore, the presence of sodium alginate, carboxymethyl cellulose solution, and calcium chloride provided protection, resulting in higher chroma and lower BI in the microcapsules compared to the single PC group. Therefore, food additives containing PC microcapsules might be favorably received by consumers due to their vibrant colors.

### 3.3. Texture

Textural analyses were conducted on the product matrix, simulating oral chewing via probe compression. Hardness is defined as the capacity of a solid material to withstand permanent deformation when subjected to compressive force [27]. The qualitative analysis indicated that the microcapsules of the composite wall material exhibited greater stiffness compared to the single sodium alginate microcapsules (*p* < 0.05) (Table 4). This suggested that the hydrogen bonding was facilitated by the hydroxyl groups on carboxymethylcellulose and the carboxyl and hydroxyl groups on the sodium alginate molecule [28]. Elasticity is defined as the capacity of a product to return to its original state after the removal of an applied external force that caused deformation. The elasticity of wet composite wall microcapsules was larger than that of wet single sodium alginate microcapsules, freeze-dried composite wall microcapsules, and freeze-dried single sodium alginate microcapsules, showing that composite wall microcapsules are more resistant to deformation (Table 4). Cohesiveness refers to the intrinsic binding force required to form the food form, gumminess indicates the energy required to chew the semisolid food into a swallowable state, while mastication reflects the energy required to chew the solid food into a swallowable state [28]. The trends in cohesiveness, adhesive properties, and chewiness between the two wall microcapsules were consistent with the trends in hardness. In summary, the mechanical properties of the microcapsules added with the two wall materials are improved.

### 3.4. Thermogravimetric Analysis

**Figure 3:**
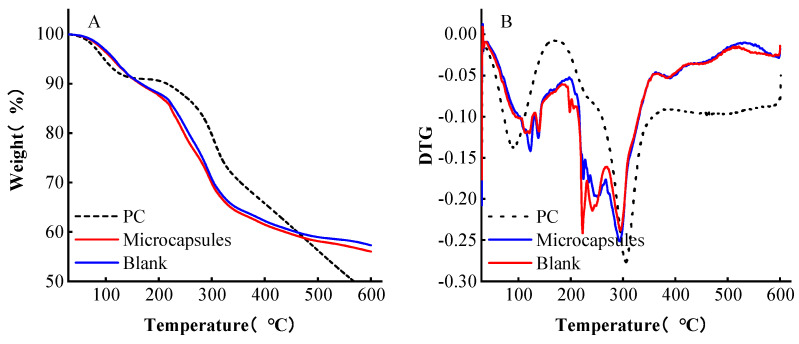
Thermostatically analysis of microcapsules. (**A**) The thermogravimetric curve (weight). (**B**) The thermogravimetric differential curve (DTG).

The thermogravimetric analysis reflects the change in sample mass with the temperature at the program-controlled temperature and is used to evaluate the thermal stability of microcapsules. From Figure 3A,B, it can be seen that the weight loss of PC was divided into two stages: 30~161 °C and 161~600 °C; the weight of PC decreased rapidly in the second stage, dropping 43.83% compared to the first stage, and lost 52.73% at 600 °C, and continuously decreased, while the weight loss of microcapsules and wall materials was divided into three stages, 30~177 °C, 177~260 °C, and 260~600 °C; the value was stable at 600 °C.

The reason for the weight loss of the three in the first stage (30~161 °C) is that the sample has a certain hygroscopicity, and the evaporation of water leads to weight loss. As seen from Figure 3B, when the original anthocyanin was violently degraded, the mass loss of the microcapsules was lower than the original anthocyanin and wall material. Additionally, the degradation temperature of microcapsules was 177 °C, which was higher than the original anthocyanin degradation temperature. As the hydrocarbon in the wall material was cleaved, the weight of microcapsules decreased in the second stage. The weight loss of microcapsules was lower than the original proanthocyanin, which proved that microcapsules are more stable than the original proanthocyanin. Interestingly, the weight loss of microcapsules occurred in the third stage as a result of the decomposition of calcium and sodium salts under the rupture of wall material. Therefore, the microcapsules were successfully encapsulated with PC and were more stable than PC alone in the temperature range of 30~600 °C.

### 3.5. UV-Vis Analysis and Fourier Infrared Spectroscopy

**Figure 4:**
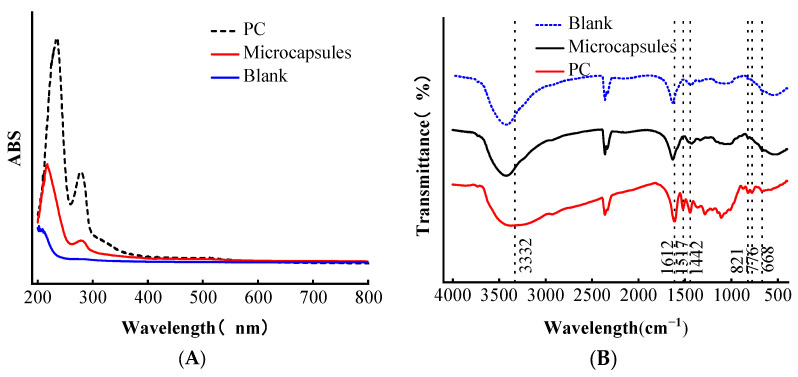
(**A**) UV full wavelength scan (ABS). (**B**) Fourier infrared spectrum (transmittance).

As shown in Figure 4A, as the benzene ring structure in PC, the PC group exhibited two characteristic absorption peaks at 238 nm and 279 nm, respectively. The produced proanthocyanidin microcapsules also showed the characteristic absorption peaks of PC at 200–400 nm, 217.5 nm, and 281 nm, respectively, and the peaks were blue-shifted compared with those of single PC. The two characteristic absorption peaks of PC were not detected in the wall material, indicating that PC was successfully encapsulated in the microcapsules.

Infrared spectroscopy shows the molecular interactions between functional groups, leading to the formation of new functional groups and or changes in existing functional groups [29], and the successful formation of microcapsules can be characterized by comparing the difference in intensity of characteristic absorption peaks before and after proanthocyanidin encapsulation [30]. The FTIR spectra of proanthocyanidin microcapsules with wall materials and PC are shown in Figure 4B.

From Figure 4B, it can be seen that there are several absorption peaks in the infrared spectrogram of PC; the broadband at 3332 cm^−1^ is caused by the stretching vibration of the -OH of the phenolic hydroxyl structure of PC [15], and the spectrograms at 1612 cm^−1^, 1517 cm^−1^, and 1442 cm^−1^ are the peaks of the benzene ring structure of PC, which are the characteristic absorption peaks of PC, 821 cm^−1^, 776 cm^−1^, 668 cm^−1^. The peaks at 821 cm^−1^, 776 cm^−1^, and 668 cm^−1^ are the CH–planar outward bending vibrations of the benzene ring of PC; only one peak at 1517 cm^−1^ and peaks in the range of 600–900 cm^−1^ indicate that the PC used in the experiment is mainly proanthocyanidin units [31]. The peaks of microcapsules and wall materials at 3423 cm^−1^ are the stretching vibration of the hydroxyl group, the peak at 1631 cm^−1^ is the symmetric stretching vibration of the carboxyl group, and the peak at 1423 cm^−1^ is the symmetric stretching vibration of -COO-. The difference between the peaks of microcapsules and wall materials in the spectra is not significant, but the peak at the broadening of the peak shape at 3423 cm^−1^ indicates enhanced hydrogen bonding, while its spectrum in the range of the characteristic absorption peak of PC is not a simple superposition of the wall material and PC, indicating that there is an interaction between the wall material and PC. The absence of the characteristic absorption peak of PC in the microcapsules represents the complete encapsulation of PC [32].

### 3.6. Stability

#### 3.6.1. pH Stability Analysis

**Figure 5:**
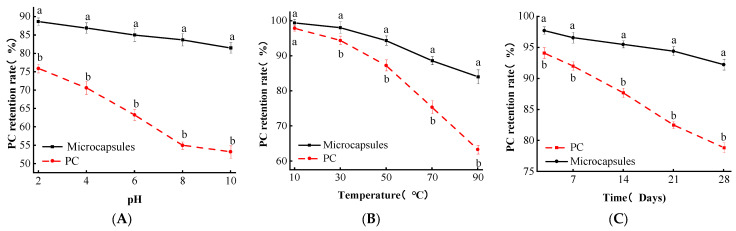
(**A**) Microencapsulation pH stability. (**B**) Microencapsulation temperature stability. (**C**) Microencapsulation storage stability. Lowercase letters (a and b) mean significant difference when *p* < 0.05.

The stability of PC and their microcapsules at different pH is shown in Figure 5A. When the pH was in the range of 2~10, the microcapsules were more tolerant of pH than a single PC at the same pH, and there was a significant difference between them (*p* < 0.05), such as that the retention of PC in microcapsules at pH 2 was 88.66%, while the retention of unembedded PC at this time was 88.66% at pH 2, and the retention rate of unembedded PC was 75.89% at this time, indicating that microcapsules enhanced the stability of PC at different pH levels to some extent.

The higher retention of PC in microcapsules may be because the wall material attenuates the effect of pH on PC to some extent. In addition, both samples exhibited a decrease in proanthocyanidin retention as the solution pH increased. This suggests that PC is more stable in acidic solutions but undergoes destructive degradation at higher pH levels.

#### 3.6.2. Temperature Stability Analysis

Heat treatment is a common operation in food processing, and the difference in PC retention in PC and microcapsules was analyzed by changing the temperature. The results are shown in Figure 5B, where the retention rate of PC in PC and microcapsules were 97.89%, and 99.41%, respectively, at 10 °C, which indicates that the temperature has a less destructive effect on both microcapsules and unembedded PC at this time. However, when the temperature increased to between 30 and 90 °C, the microcapsules were more resistant to the temperature than single PC at the same temperature, and there was a significant difference between them (*p* < 0.05), which indicates that the microcapsules enhanced the temperature stability of PC to some extent.

The high retention of PC in microcapsules may be because PC was embedded in the wall material, which blocked the effect of temperature on PC to some extent. The decrease in proanthocyanidin retention was observed in the temperature range of 30~90 °C, indicating that PCs were more stable below 30 °C but would be damaged and degraded at higher temperatures.

#### 3.6.3. Storage Stability Analysis

The proanthocyanidin retention rates of single PC and microcapsules during 28 d of storage are shown in Figure 5C. In the storage time range from 3 to 28 d, the retention rate of PC and microcapsules showed a decreasing trend, and microcapsules were more resistant to storage than single PC; there was a significant difference between them (*p* < 0.05), such as when the storage time was extended to 28 d. The retention rate of single PC decreased to 78.32%, and the retention rate of PC in microcapsules was 80.45%, which was much higher than that of single PC. The retention rate of PC in microcapsules was 80.45%, which was much higher than that of a single PC. Therefore, the prepared PC microcapsules could extend the storage time of the PC.

The retention rate of PCs in microcapsules was consistently higher than that of a single PC as the storage time increased. This is because the presence of wall materials in microcapsules weakened the damage to the PC structure caused by external factors such as air, temperature, and other environmental conditions. Microcapsules provide a certain protective effect on unstable PCs, allowing them to be preserved for a longer period.

#### 3.6.4. Metal Ion Stability

**Figure 6:**
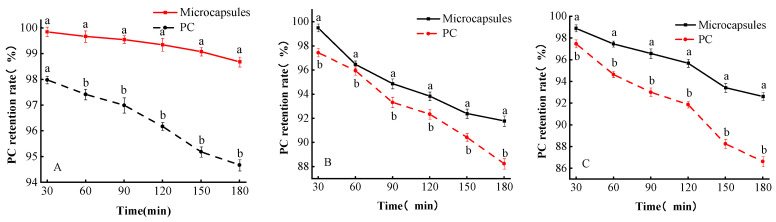

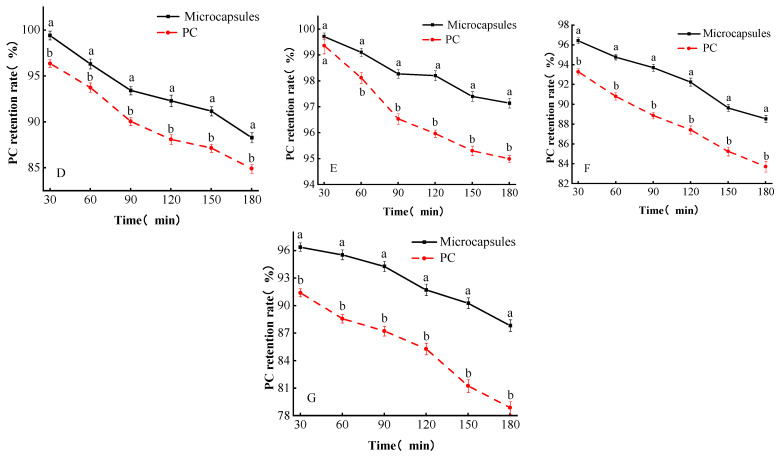
Microencapsulation metal ion stability. (**A**–**G**) show the effects of Na^+^, K^+^, Mg^2+^, Zn^2+^, Ca^2+^, Fe^2+^, and Fe^3+^ on the retention of proanthocyanidins in microcapsules, respectively. Lowercase letters (a and b) mean significant difference when *p* < 0.05.

Compounds with metal ions are often added to food products to stabilize the chemical composition, and the right amount of metal ions is also a trace element to maintain human health [33]. The effect of different metal ions on the stability of single PC and microcapsules is shown in Figure 6.

Sodium chloride is often used as a stabilizer in food processing to extend the shelf life of foods. And it is also used to improve the taste and flavor of foods [24]. As shown in Figure 6, the effect of Na^+^ on the retention of PC in microcapsules was investigated by treating PC with sodium chloride. At 30 min, the retention of PC in single PC and microcapsules was 97.97% and 99.84%, respectively, indicating that Na^+^ had little damaging effect on microcapsules and single PC at this time, but when the time was extended to the range of 60–180 min, microcapsules were consistently more tolerant to Na^+^ than single PC as time was extended (*p* < 0.05), for instance, at 90 min, the retention of single PC was 96.98%, while the retention of PC in microcapsules was 99.54% at this time. Therefore, microcapsules were more stable than a single PC when exposed to a NaCl solution treatment for 60–180 min.

The effects of K^+^, Mg^2+^, Zn^2+^, Ca^2+^, Fe^2+^, and Fe^3+^ on the retention of microcapsules and single PC are shown in Figure 6. The microcapsules were consistently more tolerant to K^+^ than a single PC from 30 to 180 min (*p* < 0.05).

The effect of Ca^2+^ on the stability of microcapsules may be the saturated cross-linking of the G-molecule chain of sodium alginate in the wall material with Ca^2+^. At the same time, carboxymethyl cellulose did not coordinate with the occurrence of Ca^2+^; therefore, the wall material forms a barrier, which protects the structure of PC to a certain extent. Flocculations appeared in the solutions of both Fe^2+^, and Fe^3+^, which was because the hydroxyl group of PCs coordinated with metal ions and generated insoluble complexes, so the retention rate of PC in microcapsules was higher because the presence of wall materials formed a physical barrier, which protected the structure of PC to a certain extent and finally showed a higher retention rate of PC. In contrast, the retention rate of PC in both PC and microcapsules showed a decreasing trend. This phenomenon might be due to the instability of PC, which affected the structure of PC due to the presence of surrounding oxygen, resulting in the hydrolysis of PC and the decrease in retention rate.

#### 3.6.5. Food Additive Stability

**Figure 7:**
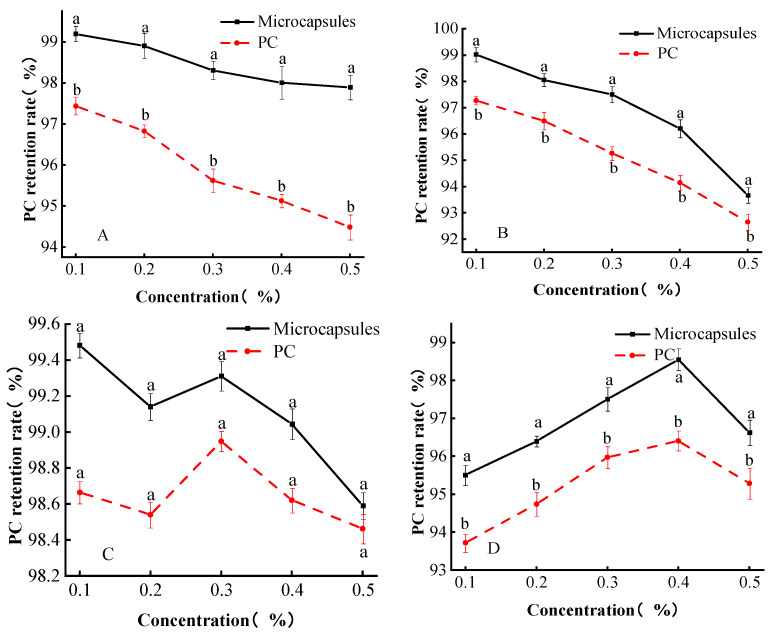
Microencapsulation food additive stability. (**A**) Treatment of microcapsules with potassium sorbate. (**B**) Treatment of microcapsules with sodium benzoate. (**C**) Treatment of microcapsules with citric acid. (**D**) Treatment of microcapsules with sodium bisulfite. Lowercase letters (a and b) mean significant difference when *p* < 0.05.

The effects of common food additives on the stability of single PC and their microcapsules are shown in Figure 7. From this, it can be seen that in the range of potassium sorbate solution concentration from 0.1 to 0.5 mg/mL, with the increase in potassium sorbate solution concentration, there was a significant difference in the retention rate of PC between the single PC and microcapsules groups (*p* < 0.05), which may be because the sodium–potassium sorbate solution was weakly alkaline to the microcapsule wall material and the PC in microcapsules (Figure 7A). The PC in microcapsules can be stabilized because of the dense protective layer formed by the sodium alginate G chain and Ca^2+^ in the wall material [14].

It can be seen in Figure 7B that in the range of 0.1–0.5 mg/mL of sodium benzoate solution concentration, the retention of PC in both single PC and microcapsules showed a significantly decreasing trend with the increase in sodium benzoate solution concentration (*p* < 0.05), indicating that sodium benzoate exhibited a certain destructive effect on both PC and microcapsules. PC in microcapsules would be more stable than the PC alone since the microcapsule wall material was stable in alkalinity compared with PC.

In Figure 7C, there was no significant difference in the retention rate of PC in single PC and microcapsules in the range of 0.1–0.5 mg/mL of citric acid solution. In the range of 0.2–0.3 mg/mL of citric acid solution, both proanthocyanidin retention showed an increasing trend, presumably because the presence of citric acid caused the hydrolysis of PC into anthocyanins, which had a certain degree of color-enhancing effect on PC [34], but it also destroyed the original structure of PC as a result, so the citric acid solution was detrimental to microcapsules and PC.

The retention of PC in both single PC and microcapsules showed a trend of increasing and then decreasing with increasing concentration of sodium bisulfite solution in the range of 0.1–0.5 mg/mL (Figure 7D) (*p* < 0.05). The decrease in proanthocyanidin retention was due to the additional reaction of excessive sodium bisulfite with PC to form colorless compounds.

The retention of PC in microcapsules was always higher than that of single PC regardless of the additives, and the reason for this phenomenon was that the G-chain of sodium alginate was cross-linked with Ca^2+^ to form a dense structure [14], while carboxymethyl cellulose filled the pores between sodium alginate molecules, both of which encapsulated procyanidins and improved the stability of procyanidins.

### 3.7. Antioxidant Activity

#### 3.7.1. DPPH Free Radical Scavenging Capacity

**Figure 8:**
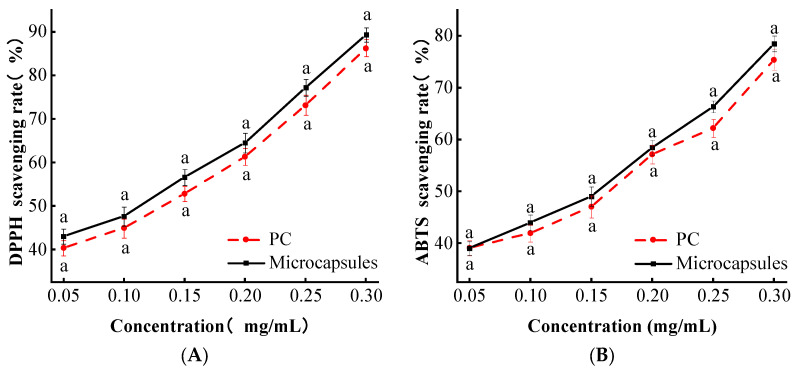
(**A**) Microencapsulated DPPH free radical scavenging capacity graph. (**B**) Microencapsulated ABTS free radical scavenging capacity graph. Lowercase letters (a) mean significant difference when *p* < 0.05.

The DPPH scavenging assay is widely used for in vitro antioxidant capacity testing and screening antioxidant fragments in natural plant extracts [7]. DPPH ethanol solutions are purple in color, and the purple hue fades when the DPPH radicals are reduced to DPPH-H upon encountering a hydrogen donor [23]. The free radical scavenging ability of DPPH from single PC and microcapsules is shown in Figure 8A. In the concentration range of 0.05–0.30 mg/mL, the scavenging ability of microcapsules and PC on DPPH free radicals was positively correlated with the concentration, while there was no significant difference between them. This could be attributed to the strong antioxidant ability of PC, whereas the antioxidant capacity of sodium alginate was considerably weaker than that of PC. The microencapsulated wall material had a synergistic effect without compromising the scavenging of DPPH free radicals by PC.

#### 3.7.2. Analysis of ABTS Radical Scavenging Ability of Microcapsules

The oxidized ABTS was able to generate stable cationic radicals of ABTS, resulting in a blue-green solution that faded after the addition of antioxidants to the reaction system. The radical scavenging ability of ABTS of both single PC and microcapsules is shown in Figure 8B. In the concentration range of 0.05~0.30 mg/mL, the ABTS radical scavenging ability of individual PC and microcapsules showed a positive correlation with the concentration. This could be attributed to the potent antioxidant capacity of PC, but there was no significant difference observed in the ABTS radical scavenging ability between PC and microcapsules. This might be because carboxymethyl cellulose in the wall material did not have the ability to scavenge ABTS radicals, and the free radical scavenging ability of sodium alginate was too low compared to PC, but it also indicated that microcapsules do not compromise the ABTS radical scavenging ability of PC.

### 3.8. Simulated In Vitro Digestion

**Figure 9:**
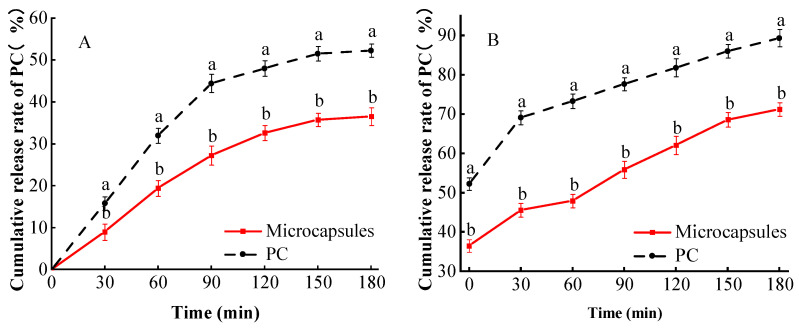
Microencapsulated in vitro simulated digestive release. The results of simulating in vitro gastric (**A**) and intestinal digestion (**B**) experiments. Lowercase letters (a and b) mean significant difference when *p* < 0.05.

The release process of microcapsules and single PC in the in vitro simulated gastric digestion is shown in Figure 9A. The cumulative release rate of PC from both single PC and microcapsules increased with increasing time from 0 to 180 min of in vitro simulated gastric digestion, and there was a significant difference between the two (*p* < 0.05). For example, at 30 min, the cumulative release rate of a single PC was 15.79%, while the cumulative release rate of microcapsules was 8.99% at this time. Figure 9A shows that during in vitro simulated gastric digestion, both substances achieved a stable release at 120 min, with the cumulative release rate of a single PC 1.47 times higher than that of microcapsules. In the in vitro simulated gastric digestion experiments, the microcapsules exhibited a lower cumulative release rate compared to the single PC. This difference may be attributed to the stability of sodium alginate and carboxymethylcellulose in simulated gastric juice, as well as the dense structure formed by both components, which attenuated the release of PC to some extent. This finding aligns with the analysis conducted by Sheng et al. [14], indicating that microcapsules have a discernible impact on the digestion of PC in the stomach. The cumulative release rate of PC was determined by in vitro simulated intestinal digestion of single PC and microcapsules, and the results are shown in Figure 9B. During 30–180 min of in vitro simulated intestinal digestion, the cumulative release rate of PC in both single procyanidins and microcapsules increased with increasing time, and there was a significant difference between them (*p* < 0.05). The release of procyanidins from microcapsules is facilitated by the stability of sodium alginate and carboxymethylcellulose in the wall material under acidic conditions of gastric juice. Subsequently, these components gradually dissolve in the simulated intestinal fluid due to the presence of solution and enzymes.

The release rate of microcapsules is found to be lower than that of a single PC, indicating that the intact and dense surface created by the microcapsule wall material hinders the entry of enzymes into the microcapsules. This phenomenon maintains disintegration resistance and leads to a slowed release of PC [35], providing a certain level of protection. These results align with the findings reported by Sheng et al. [14].

### 3.9. Microcapsule Release Kinetics

In various intervals of the identical index, the release rate constant k of microcapsules exhibited an inverse relationship with microcapsule stability [36]. Therefore, calculating the release rate constant k of microcapsules can verify the optimal conditions for microcapsule stability. As shown in Table 5, the study investigated the release kinetics of microcapsules under varying pH levels (4, 6, and 8) and temperatures (4 °C, 25 °C, and 37 °C) via model fitting.

When *n* = 0.54, it corresponds to the diffusion-limited kinetic reaction, and when n = 1, it corresponds to the primary kinetic reaction [36]. The microcapsules demonstrate good conformity with the regression equation across various temperatures and pH levels. The release of microcapsules at 4 °C, 37 °C, and at pH 4, 6, and 8 is situated between the diffusion-limited kinetic reaction and the first-order kinetic reaction. The magnitude of the release rate constant k of microcapsules at different temperatures in Table 5 was 4 °C < 25 °C < 37 °C, i.e., microcapsules were most stable at 4 °C, while the magnitude of the release rate constant of microcapsules at different pH conditions was pH 4 < pH 6 < pH 8, i.e., microcapsules were relatively stable at pH 4.

## 4. Conclusions

Microcapsules of procyanidin were effectively produced using sodium alginate and carboxymethyl cellulose. Evaluation of the fundamental physical characteristics of the microcapsules indicated favorable properties and particle structure. The advantageous flowability and stacking density of the microcapsules are conducive to industrial-scale production. The microcapsules were characterized to confirm the successful encapsulation of procyanidins, resulting in improved stability without compromising the antioxidant activity of the procyanidins. The prepared microcapsules exhibit a favorable sustained-release effect, offering a safe and effective method for incorporating PC in the food and pharmaceutical industries. So far, the limited range of suitable encapsulant materials and customer needs and preferences are still the main challenges in the future when applying these microcapsules to foods and medicines. To meet customer needs and avoid export barriers, encapsulants composed of animal derivatives, allergens, and GMOs are encouraged to be replaced by natural plant-derived ingredients.

## Figures and Tables

**Table 1:** Physical properties of lyophilized proanthocyanidin microcapsules.

Indicators	Results
Grain size (mm)	0.82 ± 0.12
Stacking density (g/mL)	0.28 ± 0.01
Firmness (g/mL)	0.32 ± 0.01
CI	13.11 ± 0.01
HR	1.15 ± 0.03
Resting angle (°)	29.87 ± 2.43

**Table 2:** Relationship between rest angle, CI, HR, and mobility of microcapsules.

Resting Angle	CI	HR	Mobility
—	≤10	1.00~1.11	Excellent
—	11~15	1.12~1.18	Very good
<30°	16~20	1.19~1.25	Good
30~45°	21~25	1.26~1.34	Good
45~60°	26~31	1.35~1.45	Fair
>60°	32~37	1.46~1.59	Poor
—	>38	>1.60	Very Poor

Note: CI represents the Carr index, and HR represents the Hausner ratio.

**Table 3:** Color analysis table. Different letters in the same row indicate a statistical difference of *p* < 0.05.

Indicators	PC	CMC	SA	Microcapsules	Blank
L*	33.46 ± 0.01 ^d^	62.22 ± 0.02 ^a^	62.21 ± 0.01 ^a^	42.07 ± 0.04 ^c^	51.84 ± 0.01 ^b^
a*	4.78 ± 0.01 ^a^	−1.56 ± 0.01 ^d^	−1.64 ± 0.02 ^d^	3.72 ± 0.02 ^b^	−0.90 ± 0.01 ^c^
b*	1.64 ± 0.02 ^e^	5.80 ± 0.02 ^c^	6.53 ± 0.02 ^a^	5.94 ± 0.02 ^b^	4.15 ± 0.04 ^d^
Chroma	5.05 ± 0.03 ^d^	6.00 ± 0.06 ^c^	6.73 ± 0.04 ^b^	7.01 ± 0.02 ^a^	4.25 ± 0.01 ^e^
Hue	0.33 ± 0.01 ^b^	−1.30 ± 0.04 ^d^	−1.32 ± 0.01 ^c^	1.01 ± 0.01 ^a^	−1.35 ± 0.02 ^cd^
BI	53.99 ± 0.01 ^a^	52.63 ± 0.03 ^c^	52.12 ± 0.03 ^d^	51.788 ± 0.02 ^e^	52.21 ± 0.01 ^b^

Note: L* indicates the brightness from black to white, a* indicates the brightness from red (+) to green (−), and b* indicates the brightness from yellow (+) to blue (−). Hue and BI indicate the Hue angle and Browning, respectively.

**Table 4:** Microencapsulated mass structure, where 1 is wet state single sodium alginate microcapsules, 2 is wet state composite wall microcapsules, 3 is lyophilized single sodium alginate microcapsules, and 4 is lyophilized composite wall microcapsules. Microcapsules are encapsulated PC, and different letters in the same column represent significant differences (*p* < 0.05).

Samples	Hardness	Flexibility	Cohesiveness	Adhesiveness	Masticatory
1	268.27 ± 10.64 ^d^	0.37 ± 0.02 ^b^	0.74 ± 0.01 ^b^	224.20 ± 14.60 ^d^	83.08 ± 1.31 ^c^
2	913.37 ± 14.21 ^b^	0.83 ± 0.01 ^a^	0.84 ± 0.03 ^a^	572.91 ± 7.23 ^a^	386.24 ± 4.95 ^a^
3	481.28 ± 7.02 ^c^	0.16 ± 0.01 ^c^	0.51 ± 0.03 ^d^	256.35 ± 22.23 ^c^	46.35 ± 1.88 ^d^
4	1310.47 ± 26.12 ^a^	0.36 ± 0.01 ^b^	0.62 ± 0.02 ^c^	535.22 ± 11.11 ^b^	257.67 ± 6.12 ^b^

**Table 5:** Release parameters of microcapsules in different environments.

Parameters	4 °C	25 °C	37 °C	pH = 4	pH = 6	pH = 8
*n*	0.5564	0.4884	0.8355	0.6241	0.7026	0.8147
R^2^	0.9621	0.9925	0.9950	0.9644	0.9874	0.9816
k	0.38 × 10^−3^	0.43 × 10^−3^	0.60 × 10^−3^	0.063 × 10^−3^	0.67 × 10^−3^	0.083 × 10^−3^

Note: *n* indicates the release mechanism parameters. R^2^ indicates the coefficient of determination. k indicates the release rate constant of the microcapsule.

## Data Availability

The article contains all the relevant data. The original contributions presented in the study are included in the article; further inquiries can be directed to the corresponding author.
